# Reduced readiness potential and post-movement beta synchronization reflect self-disorders in early course schizophrenia

**DOI:** 10.1038/s41598-021-94356-5

**Published:** 2021-07-22

**Authors:** Francesco Luciano Donati, Matteo Fecchio, Davide Maestri, Mattia Cornali, Chiara Camilla Derchi, Cecilia Casetta, Maristella Zalaffi, Corrado Sinigaglia, Simone Sarasso, Armando D’Agostino

**Affiliations:** 1 Department of Health Sciences, University of Milan, Ospedale San Paolo, Blocco A, Piano 9. Via Antonio di Rudinì, 8, 20142 Milan, MI Italy; 2 Department of Psychiatry, University of Pittsburgh, Pittsburgh, PA USA; 3 Department of Biomedical and Clinical Sciences ‘L. Sacco’, University of Milan, Padiglione ‘LITA’, Piano 5, Via Gian Battista Grassi, 74, 20157 Milan, MI Italy; 4 Center for Neurotechnology and Neurorecovery, Department of Neurology, Massachusetts General Hospital, Boston, MA USA; 5 IRCCS Fondazione Don Carlo Gnocchi ONLUS, Milan, Italy; 6 Department of Psychosis Studies, Institute of Psychiatry, Psychology and Neuroscience, King’s College London, London, UK; 7 National Psychosis Service, South London and Maudsley NHS Foundation Trust, London, UK; 8 Department of Philosophy, University of Milan, Milan, Italy

**Keywords:** Neuroscience, Psychology, Biomarkers

## Abstract

Disturbances of conscious awareness, or self-disorders, are a defining feature of schizophrenia. These include symptoms such as delusions of control, i.e. the belief that one’s actions are controlled by an external agent. Models of self-disorders point at altered neural mechanisms of source monitoring, i.e. the ability of the brain to discriminate self-generated stimuli from those driven by the environment. However, evidence supporting this putative relationship is currently lacking. We performed electroencephalography (EEG) during self-paced, brisk right fist closures in ten (M = 9; F = 1) patients with Early-Course Schizophrenia (ECSCZ) and age and gender-matched healthy volunteers. We measured the Readiness Potential (RP), i.e. an EEG feature preceding self-generated movements, and movement-related EEG spectral changes. Self-disorders in ECSCZ were assessed with the *Examination of Anomalous Self-Experience* (EASE). Patients showed a markedly reduced RP and altered post-movement Event-Related Synchronization (ERS) in the beta frequency band (14–24 Hz) compared to healthy controls. Importantly, smaller RP and weaker ERS were associated with higher EASE scores in ECSCZ. Our data suggest that disturbances of neural correlates preceding and following self-initiated movements may reflect the severity of self-disorders in patients suffering from ECSCZ. These findings point towards deficits in basic mechanisms of sensorimotor integration as a substrate for self-disorders.

Schizophrenia (SCZ) is a highly debilitating mental illness that affects up to 1% of the global population^1^. Clinically, psychotic symptoms classified as positive (hallucinations, delusions) and negative (blunted affect, apathy) are accompanied by a progressive decline in cognitive abilities and social functioning. After illness onset, typically occurring between 15 to 30 years of age, patients often experience a non-remitting, chronic-relapsing clinical course, making SCZ one of the leading causes of disability worldwide^2^.

A disturbed self-experience has been considered a core feature of SCZ since the first conceptualizations of this diagnosis^3,4^. In patients with SCZ, Self-Disorders (SDs) underlie a wide spectrum of anomalous subjective experiences, from a distortion in first-person perspective and diminished integrity of one’s subjectivity or self-world boundary to full-fledged delusions. Typical delusional themes include thought insertion, i.e. the belief that one’s thoughts are inserted by others, and delusions of control, i.e. the belief that one’s movements and behaviors are controlled by an external source^5–8^. SDs are highly specific for the SCZ spectrum^9,10^, relatively stable over time (“trait-like”)^11^, and can predict the onset of psychosis in at-risk individuals^12^. Furthermore, SDs are positively correlated with clinical predictors of poor outcome such as social dysfunction^13^, suicidality^14^, and lack of insight^15^. Therefore, SDs have recently emerged as a promising target for research that can bridge subtle modifications of subjectivity in the psychosis prodrome to the clinical outcome of SCZ in affected individuals^16^.

Recent efforts to outline the neurocognitive bases of SDs provided a framework linking disturbances of Self-experience to aberrant salience^17^ and, importantly, deficits in source monitoring^18^. Impaired source monitoring indicates difficulties in distinguishing self-generated stimuli (for example, proprioceptive inputs following a self-initiated movement) from those caused by external agents. Based on Feinberg’s seminal observations in SCZ^19^, Frith’s *comparator model* is a well-accepted theoretical blueprint for understanding self-monitoring and its disorders^20,21^. Source monitoring is believed to arise from mechanisms of neural integration occurring within the sensorimotor system. According to the model, the neural plan processed by motor areas is compared with the perceived effects in the sensory cortex (sensory reafference), thus confirming the self-generated nature of the movement. In turn, this determines the dampening of self-generated proprioceptive stimuli and sustains the subjective experience of having caused the movement (or s*ense of agency*). The neural bases of this framework postulate that an efference copy (EC) of the motor act, carrying information on the sensory effects expected from the planned movement, is transmitted to sensory areas (resulting in a “corollary discharge”, CD) where it is compared to the afferent sensation resulting from the planned movement^22^. Interestingly, several studies have shown deficits consistent with EC/CD dysfunctions in SCZ. For instance, EC/CD are affected in the oculomotor system^23^, during talking and inner speech in SCZ patients^24^. These mechanisms could provide a unique translational bridge to explore underlying neurophysiology on one side, and subtle motor abnormalities observed in patients on the other^25^.

Empirical support to the comparator model is provided by the so-called “intentional binding” phenomenon, i.e. the fact that, in healthy subjects, self-generated actions and their sensory consequences are subjectively perceived closer in time than they actually are, for example when hearing a tone triggered by a self-paced button press^26^. It was recently shown that the intentional binding is altered in SCZ, particularly in its predictive component: as opposed to healthy subjects, patients with SCZ did not show a stronger temporal binding when their button press was more likely to deliver a tone, compared to conditions where the occurrence of the tone was less likely^27^. This suggests a deficit of internal predictive mechanisms in SCZ^27,28^. In addition, and importantly, source monitoring deficits have been associated with delusions of control across several experimental paradigms^29–31^. In light of the comparator model, these findings indicate that altered mechanisms of forwarding and integrating sensorimotor information are an important feature of SCZ that might contribute to determining SDs.

The exquisite temporal resolution of electroencephalographic (EEG) recordings allows mapping brain activity on a millisecond timescale, making it a useful tool to study the neurophysiological mechanisms underlying these phenomena. The Readiness Potential (RP) is a slow brain wave that ramps up to two seconds before a self-generated movement and reaches a negative peak at movement onset^32^. Being absent before reflex, automatic, or provoked (e.g. TMS-induced) movements^32^, the RP is believed to reflect planning, preparation, and initiation of motion. Interestingly, higher-amplitude RPs have recently been associated with stronger intentional binding, suggesting that source monitoring is enhanced in subjects with larger premovement activity^33^. Conversely, the RP is known to be reduced and to have an increased onset latency in chronic, medicated SCZ patients compared to healthy control subjects^34^, and its impairment has been associated with worse psychotic symptoms^35^. However, unlike other event-related EEG potentials that have been investigated thoroughly in psychosis^36–38^, it is unclear whether a reduction of RP represents a reliable neurophysiological feature of SCZ or rather a simple by-product of neurodegenerative processes or exposure to psychotropic medications. In particular, it is not known whether the RP is altered since early in the course of SCZ.

Another important EEG feature associated with movement is the amplitude modulation of mu (10–13 Hz) and beta (14–24 Hz) frequency bands^39^. Activity in mu and beta bands is believed to represent the EEG correlate of the motor cortex activity at rest. During movement preparation and execution, mu and beta activity over sensorimotor areas shows a desynchronization (event-related desynchronization, ERD) beginning as early as 2 s before the movement and lasting until after the movement is completed^40^. This movement-related mu and beta ERD is interpreted as the functional signature of the activation of motor areas. Following this ERD, while mu spectral power returns to baseline, the beta rhythm typically shows a marked rebound. This is known as post-movement event-related synchronization (ERS)^41^. Beta ERS is recorded contralaterally to the moved effector^42^ after both executed and motorically-imagined movements^39,43^. Interestingly, post-movement ERS was also reported after passive movements but is absent during ischemic peripheral nerve block^44^. Altogether, this evidence suggests that the post-movement ERS in the beta band depends on both movement preparation and somatosensory reafference. Based on these assumptions, beta ERS has been proposed to reflect the integration of internal feedforward estimations and sensory reafferent stimuli^45^. Interestingly, one recent study employing magnetoencephalography (MEG) found that the modulation of beta rhythms following self-generated movements correlated with the sensory attenuation for a sound caused by that same movement (a button press)^46^. This likely reflects that the post-movement ERS is related to prediction and attenuation of self-generated stimuli. Importantly, other recent MEG studies have shown that the post-movement rebound in beta frequencies is reduced in the SCZ spectrum^47,48^. However, whether a relationship exists between this altered post-movement neural activity and impaired premovement potentials (such as the RP) in SCZ remains an open question.

Although theoretical links between neurophysiological abnormalities and SDs are commonly discussed, empirical evidence bridging specific neural dynamics with anomalous self-experiences in SCZ is lacking. One recent study showed that SCZ patients have abnormally prolonged temporal integration of neural activity during a self-referential task, which correlates with the severity of SDs^49^. However, the relationship between SDs and neural features of self-generated movement and sensorimotor integration is yet to be explored.

This study aimed to investigate brain activity related to self-paced movement in SCZ and to assess the association between neurophysiological correlates of self-initiated motion and SDs. In order to minimize confounding factors related to prolonged pharmacological treatment^50^, comorbidities, length of psychiatric history, and neurodegenerative processes commonly associated with SCZ^51^, we selected patients in the early stage (< 5 years) of the disorder (Early Course SCZ, ECSCZ). Our aims were to (1) investigate whether the RP is reduced since early in the course of SCZ; (2) identify abnormalities in post-movement EEG dynamics consistent with defective sensorimotor integration in ECSCZ and (3) explore the relationship between altered neural activity associated with self-generated movement and disturbances of the minimal-Self as quantified by a specific clinical rating instrument, i.e. the Examination of Anomalous Self-Experience (EASE) interview^52^.

We recruited a sample of 10 ECSCZ patients and an equal number of age- and gender-matched healthy control subjects (HC, Table 1). All subjects underwent scalp EEG recordings and right *first dorsal interosseous* muscle electromyography (EMG) while performing a simple, self-paced brisk fist closure task. The RP and the amplitude modulation of mu and beta rhythms (i.e. mu and beta ERD/ERS) were analyzed and compared between groups using nonparametric statistics (Wilcoxon rank-sum tests). Subjects with ECSCZ were evaluated for psychotic symptoms using the Positive and Negative Symptoms Scale^53^ and for SDs using the EASE interview. The dose of antipsychotic medications prescribed at the time of EEG recordings was quantified in olanzapine-equivalents. We used non-parametric correlative analyses (Kendall’s tau, t) to explore associations between neurophysiological parameters (i.e. RP slope and post-movement ERS) and clinical variables in the ECSCZ group. Please refer to the Methods section for additional details on study design and data analysis.

**Table 1:** Participants’ demographics and psychopathological evaluations.

	ECSCZ patients	Healthy controls	*P*-value
**Gender** (M:F)	9:1	9:1	1.0
**Age**	25.3 (4.0)	27.6 (4.3)	n.s
Duration of illness	1.3 (0.73)	n.a	–
Antipsychotic drugs	17.03 (7.24)	n.a	–
**PANSS scores**			
PANSS positive	19.3 (7.2)	n.a	–
PANSS negative	26.3 (9.4)	n.a	–
PANSS global	45 (14.0)	n.a	–
PANSS total	90.7 (27.0)	n.a	–
**EASE scores**			
EASE-1	26.8 (13.3)	n.a	–
EASE-2	29.9 (13.0)	n.a	–
EASE-3	5.9 (6.4)	n.a	–
EASE-4	5.1 (5.0)	n.a	–
EASE-5	10.6 (5.2)	n.a	–
EASE-10	16.4 (10.6)	n.a	–
EASE-total	78.3 (32.3)	n.a	–

Mean values (standard deviation) are reported. Age and duration of illness are expressed in years. Dosage of antipsychotic drugs at the time of electroencephalographic (EEG) recording is expressed in olanzapine equivalents.

## Results

### Despite preserved motor output, the readiness potential is greatly reduced in ECSCZ

The normalized area-under-the-curve of grand-averaged EMG signals did not show significant differences between ECSCZ and HC (ECSCZ: 70.65 μV^2^ ± 56.12 μV^2^; HC: 76.94 μV^2^ ± 26.60 μV^2^; *p* = 0.2413, Wilcoxon rank-sum test). Thus, muscular fiber recruitment and strength, although indirectly measured, were comparable between the two groups.

**Figure 1:**
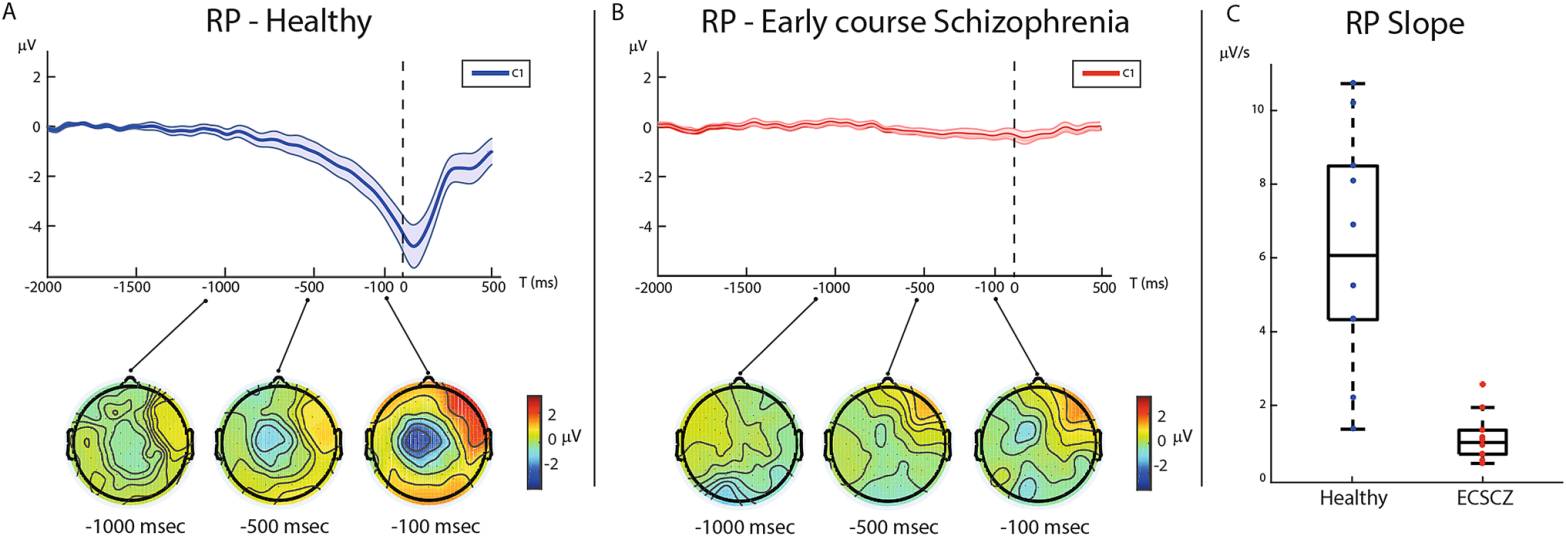
The Readiness Potential (RP) is greatly reduced in early-course Schizophrenia (ECSCZ). Time course of grand averages of electroencephalographic (EEG) trials at C1 electrode is shown for healthy subjects (**A**, blue line) and ECSCZ patients (**B**, red line). Standard errors of the mean are visualized in shaded colors. Topographic plots show scalp activity at  − 1000 ms,  − 500 ms, and  − 100 ms. Box-plot with scatter points shows little overlap in RP slope between groups (*p* = 0.004, Wilcoxon rank-sum test), corresponding to a large effect size (Cohen’s *d* = 2.18).

The RP was markedly reduced in ECSCZ patients compared to HC. RP grand averages for HC (A) and ECSCZ patients (B) are shown in Fig. 1. Standard errors of the mean are visualized in shaded colors. Box-plots with scatter points for RP slope are shown in Fig. 1C. Mean slope of the RP in the HC group was 6.18 μV/s (± 3.21 μV/s) while mean slope in ECSCZ patients was 1.12 μV/s (± 0.68 μV/s, p = 0.0004; HC: 1.35–10.73 μV/s; ECSCZ: 0.42–2.57 μV/s). The mean Maximum Peak Amplitude (MPA) of the RP in HC was -3.18 μV (± 1.66 μV) while the mean MPA in patients was -0.5 μV (± 0.60 μV, p = 0.0028). RP slope and MPA showed similarly large effect sizes (slope: *d* = 2.18 ; MPA: *d* = -2.14).

Consistent with previous findings^35^, we found a negative correlation between the RP slope and the Positive Symptoms (τ = -0.58; p = 0.0248) and Total (τ =  − 0.51; *p* = 0.0466) scores of the PANSS. No correlation was found with PANSS Negative symptoms scores (τ =  − 0.27; *p* = 0.3232). RP slope and PANSS General symptoms scores showed a trend towards a significant negative correlation (τ =  − 0.49; *p* = 0.0593). No significant correlation was found between antipsychotic doses and the RP slope (τ =  − 0.32; *p* = 0.2392).

### The post-movement ERS in the beta band is significantly reduced in ECSCZ

**Figure 2:**
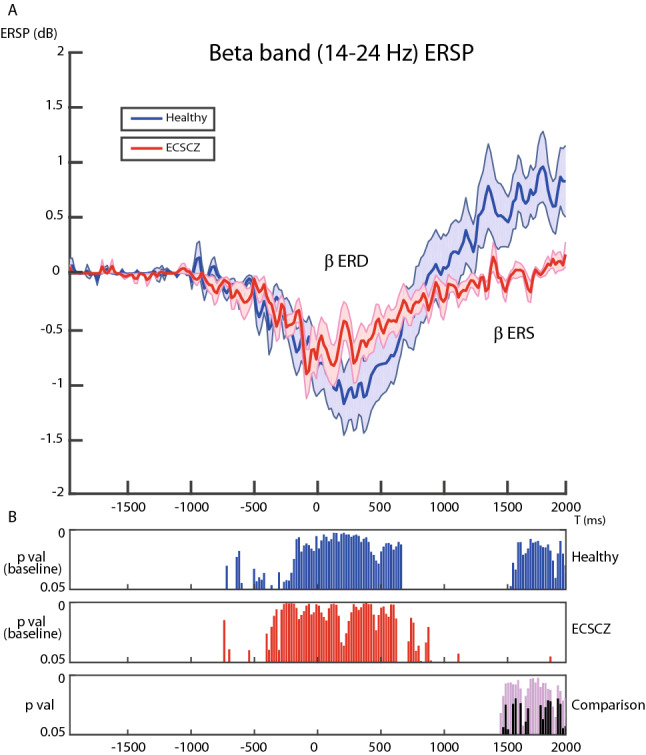
The post-movement event-related synchronization in the beta band is significantly reduced in patients. (**A**) shows the profile of the averaged event-related spectral perturbation (ERSP) for the beta band (14–24 Hz) over time for healthy controls (HC, blue line) and early-course Schizophrenia patients (ECSCZ, red line) calculated at electrodes C1-FC1. Standard errors of the mean are visualized in shaded colors. In (**B**) (top, mid), the ERSP in each time bin is compared with the baseline within each group (HC: blue bars; ECSCZ: red bars). In (**B**) (bottom) each time bin is compared between groups. Purple bars show time bins with significantly different ERSP between patients and healthy subjects (*p* < 0.05, Wilcoxon rank-sum tests). Black bars show significantly different time bins after FDR correction. While both groups showed a similar movement-related desynchronization pattern (event-related desynchronization, ERD), patients’ post-movement rebound in beta frequencies (event-related synchronization, ERS) was significantly reduced in 27 consecutive time bins (1446 to 2000 ms), of which 17 survived after correcting for multiple comparisons.

Figure 2A shows the profile of the averaged Event-Related Spectral Perturbation (ERSP) for the beta band (14–24 Hz) over time for HC (blue line) and ECSCZ patients (red line) recorded at C1-FC1. Standard errors of the mean are visualized in shaded colors. In Fig. 2B the ERSP is compared time-bin-by-time-bin to the baseline (− 2000 to − 1500 ms) for each group and between groups. Both groups showed a similar movement-related desynchronization pattern (ERD, see also Supplementary Fig. S3). However, patients’ post-movement rebound of beta frequencies (beta ERS) was significantly reduced in 27 consecutive time bins (1446–2000 ms, Fig. 2, bottom, purple shaded bars). 17 of these time bins survived after correction for multiple comparisons by False Discovery Rate (FDR, Fig. 2, bottom, black bars).

EEG activity in the mu (10–13 Hz) frequency band appeared comparable between the two groups. In particular, in both groups, the mu rhythm showed the physiological ERD and no post-movement ERS (Supplementary Fig. S4).

No correlation was found between reduced ERS and PANSS values (Positive symptoms: τ =  − 0.45 *p* = 0.0880; Negative Symptoms τ =  − 0.13 *p* = 0.6534; General symptoms τ =  − 0.40 *p* = 0.1268; Total score τ = -0.38 p = 0.1557) or between ERS and antipsychotic doses (τ =  − 0.42; *p* = 0.1197).

### Reduced RPs and post-movement ERS correlated with higher EASE scores

**Figure 3:**
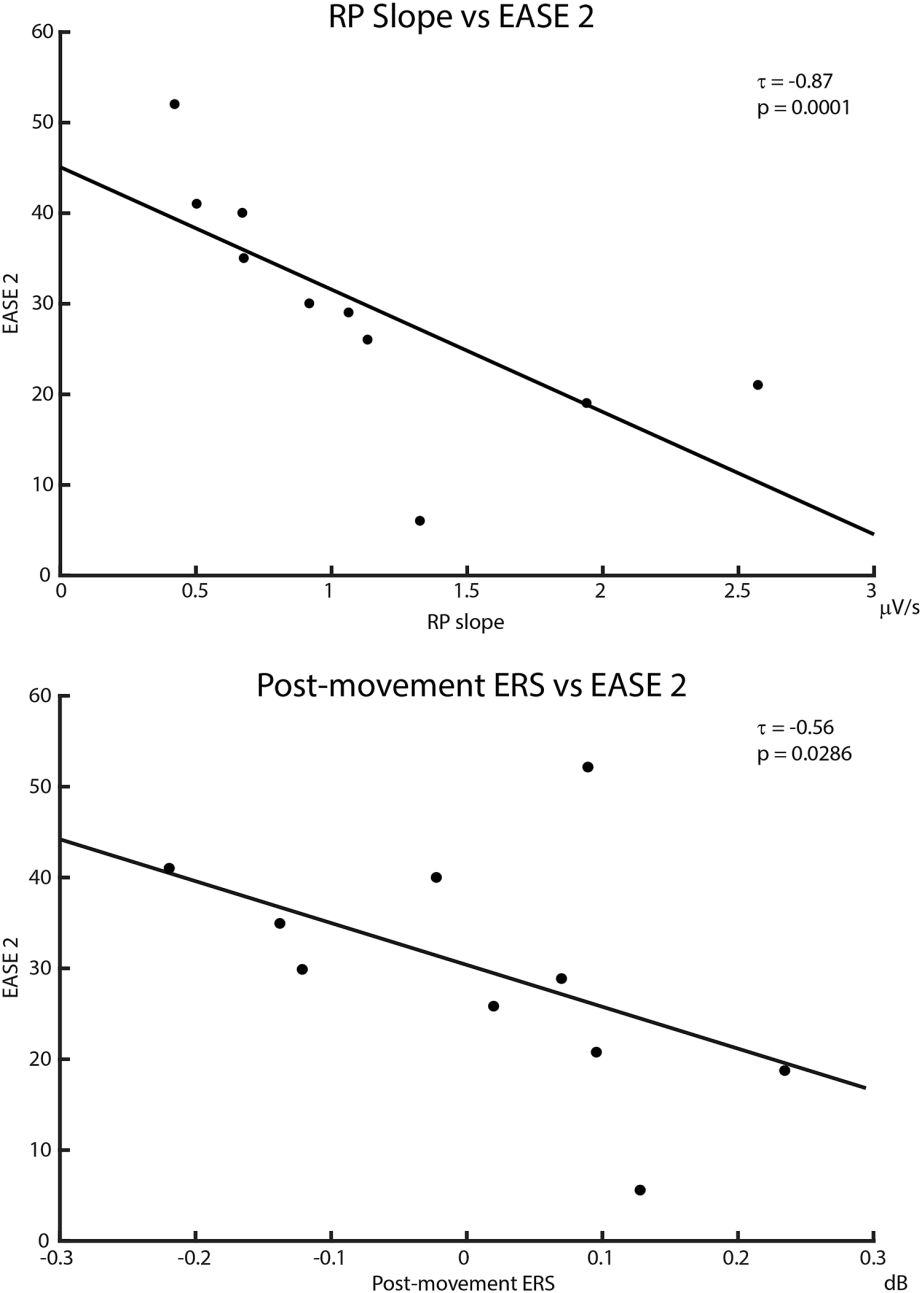
Smaller Readiness Potential (RP) and weaker post-movement Event-Related Synchronization (ERS) are associated with disturbances of self-awareness. Figure shows correlations between movement-related electroencephalographic parameters (i.e. RP and ERS) and abnormalities of self-awareness as quantified by the Examination of Anomalous Self-Experience (EASE, item 2). RP slope (top) and ERS (bottom) correlated negatively with EASE-2 scores. Correlation coefficients (Kendall’s tau, τ) and significance (*p*-values) are shown.

Table 2 shows correlations coefficients (Kendall’s tau, τ) between EASE subitems scores and the movement-related neurophysiological parameters that were reduced in patients, i.e. the pre-movement RP (slope) and post-movement beta ERS. The RP slope correlated negatively with EASE-2 (τ =  − 0.87; *p* = 0.0001), EASE-10 (τ =  − 0.65; *p* = 0.0116), and total EASE score (τ =  − 0.64; *p* = 0.0091). Patients’ cumulate ERSP in those 17 time bins where beta ERS was significantly reduced after FDR correction, compared to healthy controls, showed a negative correlation with EASE-2 (τ =  − 0.56; *p* = 0.0286) and EASE-5 (τ =  − 0.57; *p* = 0.0305). Similar results were obtained when cumulating ERSP values from all 27 non-FDR corrected time bins (Supplementary Table S2). Figure 3 shows scatter plots with trend lines for correlations between neurophysiological parameters and EASE-2. Similar plots for the remaining significant correlations are shown in Supplementary Figs. S5, S6. Interestingly, in the ECSCZ group, RP slope and ERS were directly correlated, although this relationship only showed a trend towards statistical significance (Pearson’s r = 0.61; *p* = 0.0584; Supplementary Fig. S7).

**Table 2:** Correlations between pre/post-movement electroencephalographic (EEG) correlates and Self-Disorders in patients.

	RP slope	Beta ERS
EASE-1 (cognition and stream of consciousness)	τ = − 0.18 *p* = 0.5296	τ = − 0.18 *p* = 0.5296
EASE-2 (self-awareness)	**τ = ** − **0.87** ***p* = 0**.0001*	**τ = ** − **0**.**56** *p* = 0.0286*
EASE-3 (bodily experiences)	τ = − 0.32 *p* = 0.2392	τ = 0.05 *p* = 0.9279
EASE-4 (demarcation	τ = − 0.23 *p* = 0.4139	τ = 0.2300 *p* = 0.4139
EASE-5 (existential reorientation)	τ = − 0.29 *p* = 0.2793	**τ = ** − **0**.**57** ***p* = 0**.0305*
EASE-10 (comprehensive)	**τ = ** − **0**.**65** ***p* = 0**.0116*	τ = − 0.25 *p* = 0.3672
EASE-total score	**τ = ** − **0**.**64** ***p* = 0**.0091*	τ = − 0.24 *p* = 0.3807

The slope of the Readiness Potential (RP) was calculated at C1 electrode in the interval  − 650 ms to − 100 ms. Beta event-related synchronization (ERS) was calculated at C1-FC1 cluster as the cumulate of the beta event-related spectral perturbation (ERSP) in time bins showing significant differences compared to healthy controls. Correlations with specific items from the Examination of Anomalous Self-Experience (EASE) are shown.

## Discussion

In this study, we used EEG recordings during a self-paced motor task to reveal a marked deficit of the RP, a well-known correlate of self-generated movement, in a sample of patients with ECSCZ (Fig. 1, Supplementary Fig. S1). No relevant abnormality in the EMG activity was observed in patients, confirming that motor output was preserved. Furthermore, we showed that, despite exhibiting unaltered movement-related mu and beta desynchronization (Fig. 2, Supplementary Figs. S3, S4), ECSCZ patients failed to show the physiological post-movement rebound in beta rhythms (beta-ERS, Fig. 2), an EEG feature that depends on both movement preparation and sensory reafference^45^. We confirmed the correlation between RP and positive symptoms, an association that had previously been suggested^35^. Most importantly, in patients, the magnitude of deficits in both pre-movement (RP) and post-movement (beta ERS) neural dynamics correlated with disturbances in minimal self as quantified by the EASE scale, particularly in the domain of self-awareness (Fig. 3, Supplementary Figs. S5, S6, Table 2). Thereby, patients with smaller RPs and weaker ERS also had more prominent SDs; conversely, patients with a relatively preserved self-experience had RP and ERS more similar to those observed in healthy subjects.

RP abnormalities have been previously reported in chronic SCZ^34,35^. However, it is unclear how early these deficits arise along the temporal trajectory of the disorder and, thereby, to what extent they might be associated with its pathophysiology. Our finding that the RP is reduced since the first stages of full-blown SCZ suggests an early dysfunction of mechanisms responsible for this neurophysiological marker.

The RP originates from a network involving the pre-supplementary motor area (pre-SMA)^32,54^, a brain area that is clearly involved in movement preparation^55^, and the anterior cingulate cortex (ACC)^56^. Effective connectivity between these two areas appears critical to sustaining the RP^57^. Evidence from subcortical recordings shows that, during the preparation of self-generated movements, electrodes recording activity from the thalamus (a structure notably altered in SCZ^58,59^) show a slow deflection in electric potential that is similar to the RP in its onset, time course, and sharp return to baseline after movement execution^60^. Interestingly, the same study showed that, during movement preparation, the SMA and the thalamus show selective coherence in the beta band, as opposed to other frequency bands^60^. Additionally, recent studies suggest that the RP is closely coupled with increased connectivity in beta and gamma frequencies in the pre-frontal cortex (PFC)^61^, and that movement intention can be predicted by analyzing these dynamics^62^. Deficits in fast oscillations (i.e. beta and gamma), particularly in the frontal lobe, are a well-known neurophysiological feature of SCZ and have been demonstrated in a number of different settings^63–65^. Structures responsible for generating these oscillations critically involve the synchronized firing of parvalbumin-positive GABAergic interneurons^66^, a neuronal population that has recently been implicated in the pathogenesis of SCZ^67,68^. We hypothesize that defects in the synchronous activity of these neurons may underlie the abnormalities in both RP generation and beta frequency post-movement rebound we observed in this study. Future research should address these observations and clarify the relationship between reduced RP and beta ERS in SCZ and altered brain dynamics underlying their generation.

Deficits in source monitoring are believed to underlie abnormalities of self-experience in SCZ, including disturbances in self-awareness^18^. Although basic neurophysiological mechanisms of source monitoring are yet to be fully uncovered, accumulating evidence points towards dynamics of sensorimotor integration based on EC/CDs^22,69^. In patients with SCZ, Ford et al. found significantly reduced phase locking of gamma (36–45 Hz) EEG activity before self-paced button presses, which failed to correlate with the somatosensory response (as seen instead in HC)^70^. In a following study, the same group showed that, in SCZ patients, the impairment in RP preceding a self-paced button press correlated with deficits in suppression of auditory N1 component for a tone delivered consequently to the button press^71^. This finding was interpreted as a failure of EC/CD mechanisms that, before movement execution, would inform the auditory cortex of the incoming sound expected after the button press. In this light, CDs during self-generated movements appear to be related to the RP, a view that is supported by the evidence that intentional binding is stronger in individuals with larger RP^33^. In this study, we found that, during a self-paced motor task, patients with ECSCZ had markedly reduced RP and impaired post-movement beta resynchronization, an EEG correlate of movement that depends at least in part on motor planning^45^ and that seemingly indexes internal predictions of the sensory consequences of one’s movements^46^. Furthermore, these deficits in RP and post-movement beta ERS were related to the severity of SDs in the ECSCZ group, although this correlation was weaker for ERS. The fact that patients with SCZ showed a reduction in neural features both preceding (RP) and following (beta ERS) self-generated movements may suggest that a relationship exists between the two phenomena. However, current evidence in healthy subjects is scarce and only partially consistent with this hypothesis^72^. Interestingly, in our study, patients showed a trend towards a significant direct correlation between RP and post-movement ERS (r = 0.61; *p* = 0.0584, Supplementary Fig. S7).

Based on our findings, a role for a failure of sensorimotor EC/CD in undermining basic mechanisms of self-experience in SCZ can only be speculated. During movement preparation, an EC of the motor plan may contribute to determining the rebound in beta frequencies seen in HCs, possibly after matching the sensory predictions with the sensory reafference generated by the execution of the planned movement. While this hypothesis will need to be specifically tested in healthy subjects, where the relationship between the RP and post-movement ERS is still poorly understood^72^, a failure of such a mechanism in SCZ might at least in part explain our findings. This speculation appears consistent with theoretical models of SDs based on altered source monitoring^19,21,27^. Indeed, altered self-awareness and symptoms such as delusions of control might originate from persistent inaccurate sensorimotor integration (i.e. abnormal “bottom-up” processing)^18^. However, the relationship between RP and post-movement ERS, both in healthy subjects and SCZ, will need to be elucidated by future research.

Beyond the relevant correlation with general measures of anomalous self-experience (EASE-10 and EASE total score), the finding of a correlation between RP/beta ERS and self-awareness (EASE-2), and the lack of relationship observed with other domains must be cautiously interpreted. Indeed, a correlation with the Bodily experiences (EASE-3) domain might have been expected. Our findings suggest that RP/ERS abnormalities reflect disruption of higher-order cognitive processing that might lead to a diminished sense of basic self or initiative rather than kinesthetic experiences or motor disturbances. However, larger patient samples are needed to clarify the relationship between observed neurophysiological abnormalities and specific domains of SDs.

SDs emerge early in the natural history of SCZ, well before positive symptoms^3,73^. Moreover, SDs are known to predict the onset of psychosis in at-risk individuals^12^. Our study reveals putative neurophysiological markers of such abnormalities that could prove useful in the clinical setting. Specifically, RP and ERS abnormalities may be associated with the motor neurological soft signs often seen in the prodromal stages of SCZ spectrum disorders^74,75^, and that were recently linked to a higher risk of a chronic course of illness after a first psychotic episode^76^. Interestingly, Poletti et al. recently argued that CD mechanisms are likely to develop in early childhood and that abnormalities in this neurophysiological domain may represent an early trace (and, putatively, a core pathophysiological mechanism) of SCZ^25^. In this perspective, the clinical relevance of RP and ERS in the prodromal stages of psychosis should be further investigated by future studies.

A limitation of this study is represented by the small size of our study sample. Although our results will need to be confirmed in larger populations, the largely non-overlapping distributions of RP parameters across the two experimental samples (Cohen’s *d* = 2.1) support the robustness and consistency of our findings. A further limitation is the substantial lack of female gender in our sample. This aspect is partially controlled by the study design, based on a gender-matched control group. One additional limitation is that our data lack direct measures of agency specific to the executed movements. Therefore, we cannot make any inference on subjects’ perceived agency over hand movements associated with the RP and ERS recorded at the scalp level. Investigating specific experimental paradigms of sense of agency, including, but not limited to, intentional binding^77^ and other indexes of sensorimotor integration known to be defective in SCZ^78^, along with neurophysiological measures, could contribute to narrowing the gap between psychopathology and biology in the study of SDs in SCZ.

Deficits in EEG features associated with planning and monitoring of self-generated movements can be observed in the early stages of SCZ and are related to abnormalities of self-experience and self-awareness. By investigating neural dynamics arising from motor preparation and sensorimotor integration in subjects affected by SCZ, we provided empirical evidence that may support the role of EC/CD mechanisms deficits in sustaining abnormal self-experience in SCZ spectrum disorders, corroborating a well-accepted yet largely unproven theoretical framework. Future research should address the potential role of such biological markers in the early recognition and stratification of SCZ spectrum disorders and at-risk mental states.

## Methods

### Participants

Ten patients (M = 9; F = 1) were recruited from our inpatient (n = 8) and outpatient (n = 2) units. An equal sample of age- and gender-matched healthy volunteers was recruited by word of mouth. Participants’ demographics and relevant psychopathological scales are shown in Table 1. Inclusion criteria for the ECSCZ group were age < 35 years, a diagnosis of SCZ according to the Diagnostic and Statistical Manual of mental disorders^79^ (DSM-5), a maximum of 5 years since the first psychotic episode, and the ability to understand and perform the task. Exclusion criteria included major medical or neurological illnesses affecting the central nervous system (CNS) or motor pathways, psychiatric comorbidities according to DSM-5, inability to understand instructions or perform the task, and any developmental disorder. No specific pharmacological treatment was deemed as an exclusion criterion for this study. Based on experts' recommendations^80^, we set a minimum threshold of 50 retained EEG trials after artifact rejection for including a subject for further analyses. Patients’ clinical assessment, including diagnosis and symptoms severity evaluation using the Positive and Negative Symptoms Scale^53^, was performed by an experienced psychiatrist. Exclusion criteria for healthy control subjects included personal or family history of any psychiatric condition according to DSM-5, any major medical or neurological illness affecting the CNS or motor pathways, and any developmental disorder. Control subjects were not undergoing any pharmacological treatment except for combined estroprogestinic oral contraceptive therapy (one subject). All subjects volunteered to participate and signed informed consent. Our local ethical committee (Milan “Area 1” Ethics Committee) approved the study protocol, and all procedures were conducted in conformity to the Declaration of Helsinki.

### Assessment of self disorders

All patients were administered the Examination of Anomalous Self-Experience (EASE) interview^52^. We selected this tool given its wide use in research studies on SDs in SCZ^10–14,49^. All patients completed the entire interview. Aggregated scores were computed for EASE domains 1 (disturbances of cognition and stream of consciousness), 2 (disturbances of self-awareness and presence), 3 (anomalous bodily experiences), 4 (demarcation deficits and/or transitivism), 5 (existential reorientation). The comprehensive ‘EASE-10’ score was also calculated including (i) hyperreflectivity (ii) loss of common sense/perplexity (iii) mirror-related phenomena (iv) loss of thought ipseity (v) spatialization of experience; (vi) ambivalence (vii) diminished sense of basic self (viii) loss of first-person perspective (ix) perceptualization of inner speech and (x) thought pressure. A summary of EASE scores is reported in Table 1 together with our participants’ demographics.

### Experimental setting

Participants sat comfortably on a chair, with eyes open, resting their right arm on a flat surface. Procedures were run during the daytime. A 64-channels EEG system (BrainAmp DC, Brain Products, Germany) was used to perform EEG recordings. 60 surface electrodes were mounted on an elastic cap (EasyCap, FMS) according to the International 10/20 System. Two electrodes recorded EOG signal and two surface electrodes recorded right *first dorsal interosseous* muscle electromyography (EMG). Electrodes were referenced to FCZ. Impedances were kept below 10 kΩ. Signal was acquired with a sampling rate of 5000 Hz. Hardware filters were set DC to 1000 Hz. Subjects were asked to perform self-paced, brisk fist closures of their right hand around a squeeze ball. Four discrete sessions were recorded for each subject. Each session had a fixed duration of ten minutes (for a total recording time of 40 min). Between sessions, subjects were allowed to rest for at least one minute. The average total n of fist closures was 258.4 ± 89.44 (SD) and 288.1 ± 104.18 (SD) for HC and patients, respectively (p = 0.5027, see Supplementary Table S1).

### Data analysis

EMG and EEG data analysis were performed using a custom Matlab (Math Works Inc. v2017a) pipeline based on the open-source toolbox EEGLAB^81^. Data were analyzed blindly to patients’ psychopathological evaluation. EMG signal underwent 40 Hz high-pass filtering (Butterworth, 4th order), rectification, peak interpolation using Hilbert transform function, and Gaussian smoothing. Amplitude and standard deviations of the EMG signals were computed, and fist closure onsets were identified using a semi-automated algorithm based on signal deviation from the electrical baseline. Movement onsets, corresponding to the rising phase of the EMG activity associated with fist closures, were extracted and used as triggers (Time 0, T0) for EEG data segmentation. Additionally, in order to detect differences in movement output between patients and control subjects, the area-under-the-curve of the EMG signal following movement onset (0–1000 ms) was computed for each subject after normalization performed by subtracting the average baseline EMG amplitude. Grand averages were then compared between the two groups (Wilcoxon rank-sum test).

EEG signal was filtered using a 0.1–80 Hz band-pass and a 49–51 Hz notch filter, then down-sampled to 1000 Hz. Each EEG session was split into trials ranging from  − 2000 ms to + 2000 ms around the movement onset (T0) extracted from EMG analysis as previously described. EEG channels showing high-frequency noise or other persistent artifacts (i.e. low-frequency drifts due to poor channel contact or sweating) were visually identified, removed, and interpolated. Given the strictly self-paced nature of the task we employed, some trials had to be excluded to avoid overlapping windows of analysis around T0. Trials showing movement artifacts were manually removed by visual inspection. The number of trials analyzed after artifact rejection was comparable between groups (HC: 173.3 ± 60.2; ECSCZ: 165.2 ± 62.8, *p* = 0.7700), corresponding to a percentage of retained EEG trials of 67.4% ± 11.03% (HC) and 58.2% ± 12.77% (ECSCZ, *p* = 0.1026, see Supplementary Table S1). Although some groups suggest excluding patients with less than 50% of retained EEG trials in studies on event-related potentials^82^, we chose not to apply a fixed threshold to this strictly self-paced experiment, in which many trials had to be rejected to avoid overlapping time windows. On the other hand, no subject had a number of retained trials < 50 (HC min: 84; ECSCZ min: 80; Supplementary Table S1). In fact, the number of trials retained for each participant was in line with, or well above, those reported by previous studies employing similar tasks in SCZ^34,35,70,71^. EEG signal was baseline corrected by subtraction of the average EEG activity between  − 2000 to  − 1500 ms and re-referenced to the average of all channels. Independent Component Analysis (ICA) was then computed. Only components clearly imputable to eye blinks and muscle activity were excluded. Finally, an 8 Hz low-pass, 3rd order Butterworth filter was applied before averaging trials, similarly to previous studies investigating the RP^83–85^. Readiness potential analysis was performed on channel C1, i.e. the scalp EEG lead exhibiting larger average RP responses in healthy control subjects (Supplementary Fig. S1). Based on previous literature, we evaluated the RP by calculating the slope of the negative drift from -650 ms to -100 ms latencies^86,87^. We also assessed the maximum amplitude of the negative peak (maximum peak amplitude, MPA) occurring between  − 200 ms and  − 100 ms. Grand averages of RP slope and maximum RP amplitude were computed for each group. Given the small sample sizes, a non-parametric test (Wilcoxon rank-sum test) was used to compare results and assess significant differences. Cohen’s *d* was used to calculate effect sizes.

On the other hand, to investigate movement-related ERD and ERS, a time–frequency decomposition was conducted using Morlet Wavelet transform (3 cycles). Frequency resolution was linearly spaced (0.0417 Hz) and time-resolution was set at 0.02 s. The event-related spectral perturbation (ERSP) was then computed between 4 and 45 Hz after adding a zero-padding of 375 ms to border values. This frequency interval was chosen to investigate typical wake EEG activity while avoiding any possible contamination from residual electric line noise. Given the sampling rate of 1000 Hz (after downsampling), wavelet length at 4 Hz was 750 samples. Absolute spectra normalization^88^ and baseline correction (− 2000 to − 1500 ms) were applied. The averaged ERSP for each group was visualized for all EEG channels (Supplementary Fig. S2). Based on previous findings^41,89^, we investigated ERD and ERS dynamics in two electrode clusters, C1-CP1 and C1-FC1 for the beta (14–24 Hz) and mu (10–13 Hz) frequency bands^39,41^. Within-group ERD and ERS were quantified by calculating significant deviances from the baseline and then compared between groups (Wilcoxon rank-sum tests). The False Discovery Rate (FDR) method was then applied for time bins following movement onset to account for multiple comparisons. In patients, the ERSP of time bins showing statistically significant differences compared to HC after FDR correction was cumulated, and the resulting value was used for correlations with clinical scales scores.

### Correlation analyses

Statistical analyses were performed in Matlab (v2017a). RP slope and ERS were correlated to antipsychotic doses, PANSS, and EASE scores with Kendall’s tau (τ) rank correlation coefficient. This non-parametric correlation test was chosen due to the negative skewness of the data distribution. Pearson’s correlation coefficient was used to correlate RP slope to ERS cumulate values.

## Supplementary Information

Supplementary Information.
