# Supplementary material for: Reduced readiness potential and post-movement beta synchronization reflect self-disorders in early course schizophrenia
Source: Sci Rep. 2021 Jul 22;11:15044. doi: 10.1038/s41598-021-94356-5 (PMC8298598; doi:10.1038/s41598-021-94356-5)
Supplement: Supplementary file 1 — Supplementary Information. [file 41598_2021_94356_MOESM1_ESM.pdf]

Reduced readiness potential and post-movement beta synchronization reflect self-disorders in early course schizophrenia.

Francesco Luciano Donati, Matteo Fecchio, Davide Maestri, Mattia Cornali, Chiara Camilla Derchi, Cecilia Casetta, Maristella Zalaffi, Corrado Sinigaglia, Simone Sarasso, and Armando D'Agostino.

<u>**Supplementary figures**</u>:

Figure S1. (**A**) All-channels display of average RP for healthy subjects (left) and ECSCZ patients (right). (**B**) shows electrodes overlying the motor cortex. Larger average RP responses were recorded contralaterally to the movement. Electrode C1 showed the larger average premovement activity in healthy subjects (top, red dotted line). Vertical lines represent movement onset.

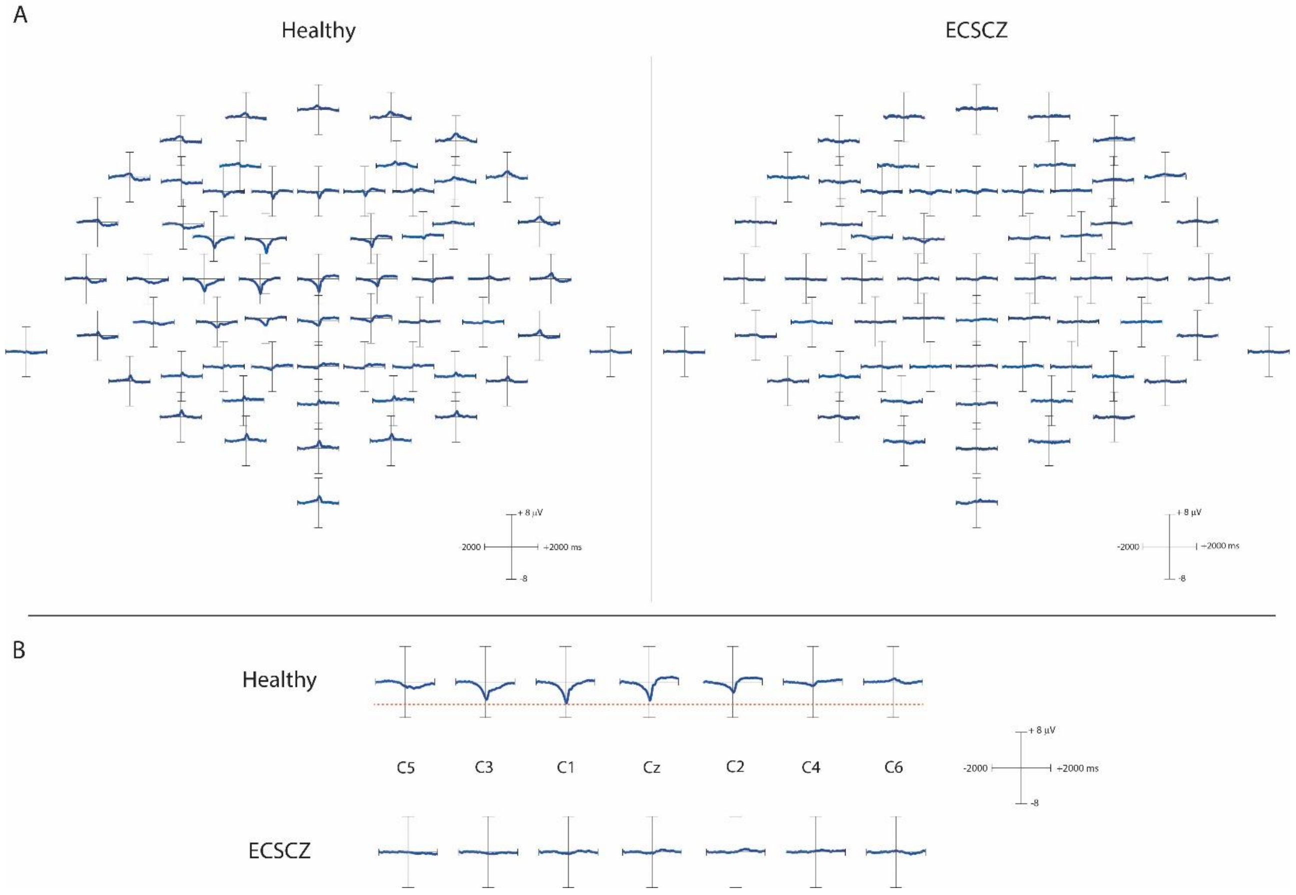

Figure S2. All-channels display of average ERSP (4-45 Hz) for healthy subjects (left) and ECSCZ patients (right). Vertical dotted lines represent movement onset.

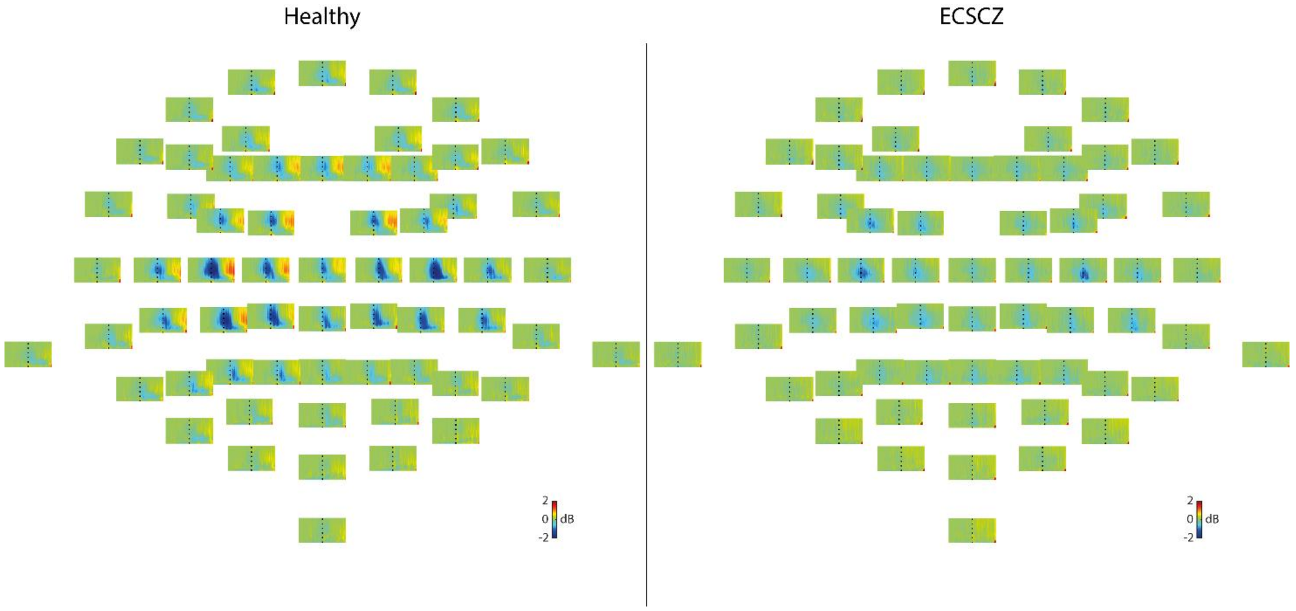

Figure S3. Time course of the ERSP in the beta frequency band recorded from C1-CP1. Consistent with previous literature[1,2], the beta ERD is more pronounced in a location slightly posterior to the coronal line, while the beta ERS is maximized in a slightly anterior location (see Figure 2). Importantly, similar to what was observed at C1-FC1, no significant differences in beta ERD were found between HC and ECSCZ patients.

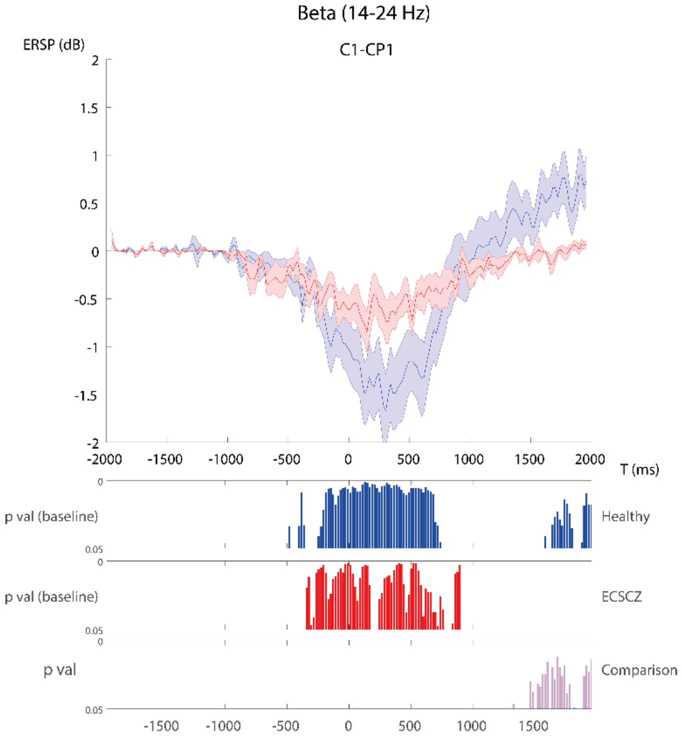

Figure S4. Time course of the ERSP in the mu frequency range recorded from electrodes C1-FC1 and C1-CP1. The mu rhythm shows a similar pattern between HC (blue lines) and ECSCZ patients (red lines). Unlike beta ERD, the mu ERD is not followed by a marked rebound in spectral power.

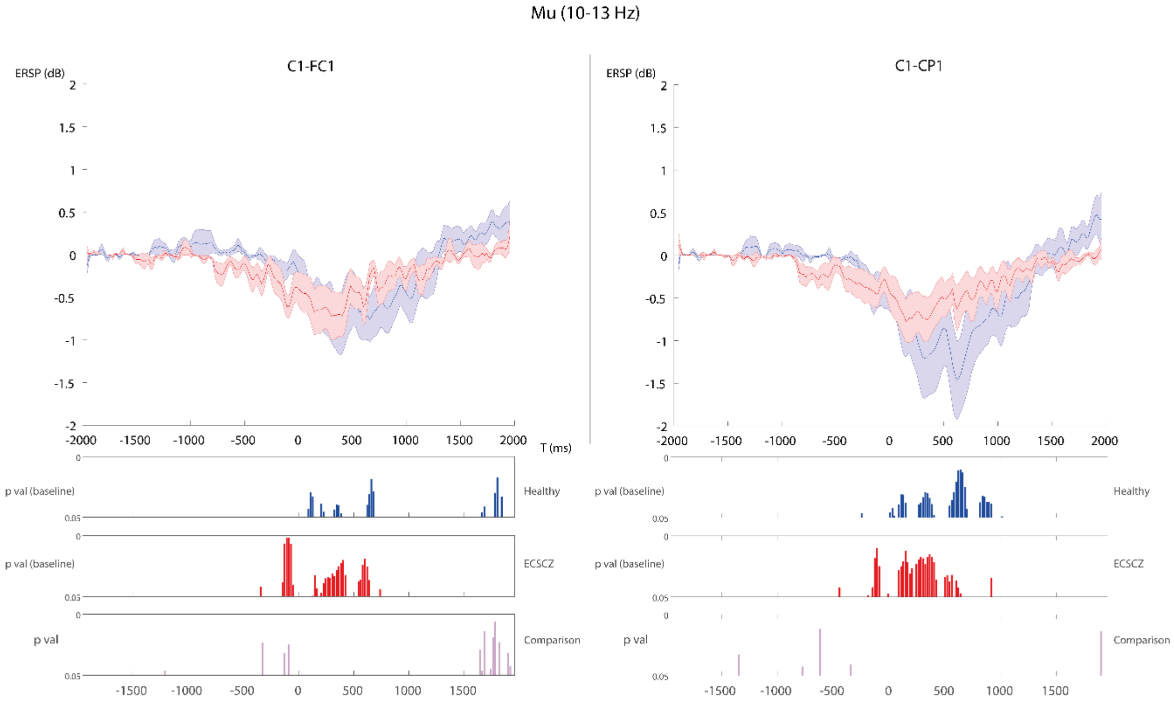

Figure S5. Correlation between RP slope and EASE 10 (top) and between RP slope and the total EASE score in SCZ patients. EASE 10 is a comprehensive item that summarizes the most prototypical aspects of Self-Disorders.

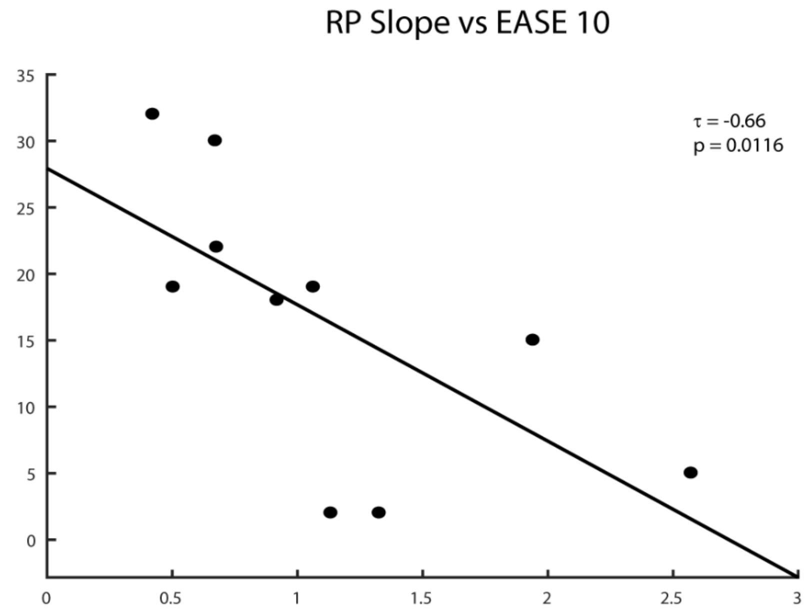

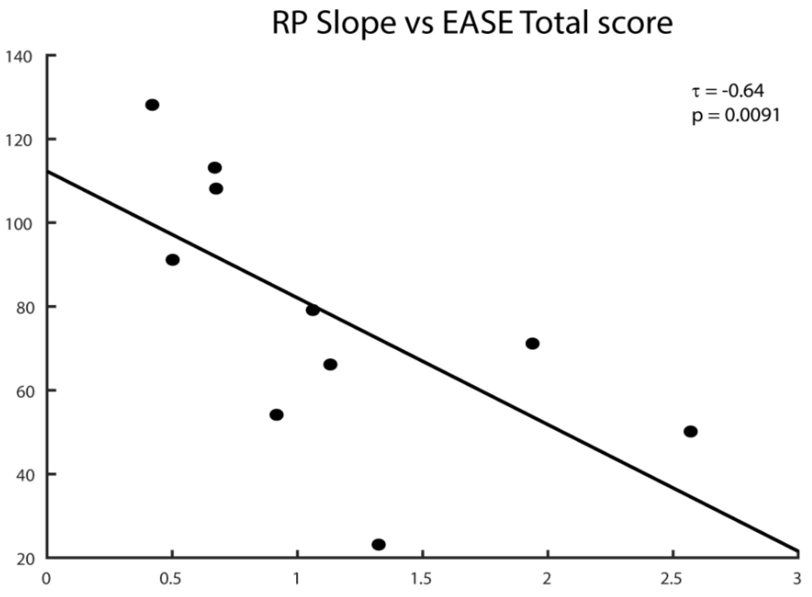

Figure S6. Correlation between SCZ patients' cumulate ERS in significantly different time bins compared to healthy controls and EASE 5 ('existential reorientation') values.

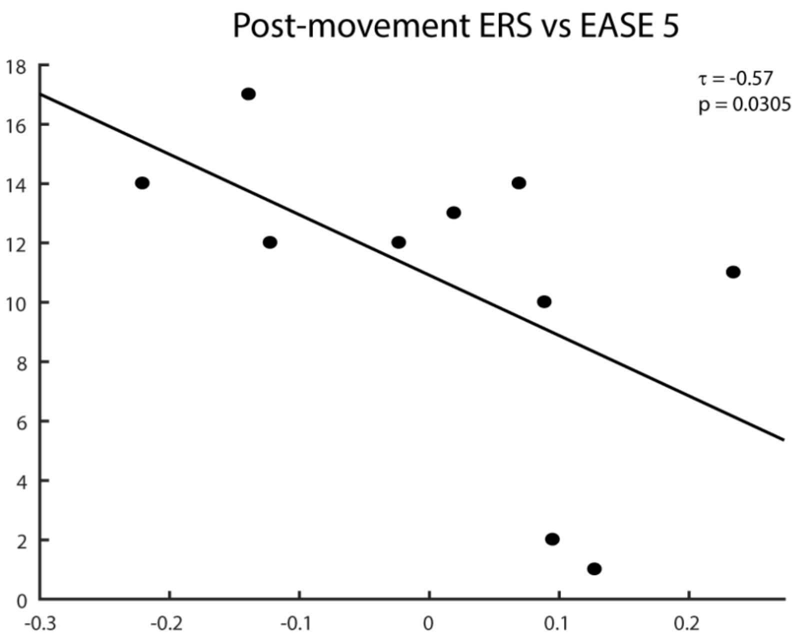

Figure S7. Correlation between the slope of the RP and beta ERS in ECSCZ patients. P-value and Pearson's r are reported.

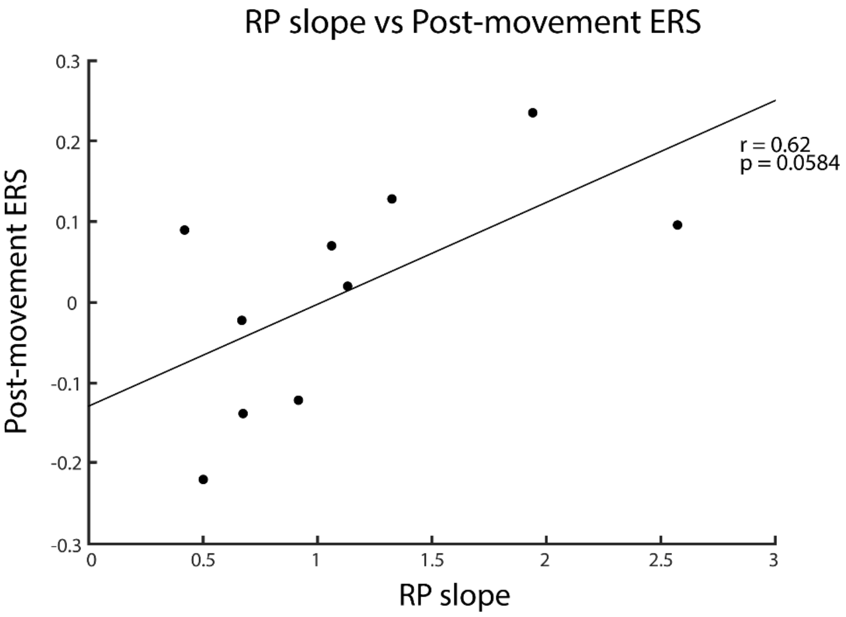

__Supplementary tables__:

Table S1. Single-subject information on the number of performed movements and retained EEG trials/channels for healthy control subjects (HC) and schizophrenia patients (SCZ). No significant differences were found between groups when comparing the number of fist closures subjects performed (i.e. N of trials, column 1, p=0.5027), the number of artifact-free EEG trials ("good EEG trials", column 2, p=0.7700), the percentage of good trials over the number of performed movements (column 3, p=0.1026), and the number of EEG channels removed during artifact rejection ("bad EEG channels", column 4, p=0.5000). Reported p values derived from unpaired t-tests. SEM: standard error of the mean.

	N of trials	N of good EEG trials	% of good EEG trials	Bad EEG channels
HC 01	145	107	73.8	0
HC 02	152	84	55.3	2
HC 03	460	236	51.3	2
HC 04	294	238	80.9	1
HC 05	232	151	65.1	1
HC 06	272	172	63.2	4
HC 07	255	165	64.7	3
HC 08	215	133	61.9	6
HC 09	246	174	70.7	1
HC 10	313	273	87.2	4
**Mean** (HC)	258.4	173.3	67.4	2.4
**SD** (HC)	89.44	60.20	11.03	1.8
SCZ 01	418	199	47.6	3
SCZ 02	449	220	49.0	2
SCZ 03	228	80	35.1	6
SCZ 04	179	130	72.6	3
SCZ 05	342	237	69.3	3
SCZ 06	234	152	65.0	0
SCZ 07	150	101	67.3	7
SCZ 08	387	254	65.6	2
SCZ 09	221	100	45.2	2
SCZ 10	273	179	65.6	2
**Mean** (SCZ)	288.1	165.2	58.2	3.0
**SD** (SCZ)	104.18	61.84	12.77	2.05
**pval**	**0.5027**	**0.7700**	**0.1026**	**0.5000**

Table S2. Kendall's τ and p values for correlations between ERS and EASE scores when cumulating all 27 statistically significant time bins for ERS before false-discovery rate (FDR) correction. As shown, results are similar to those after FDR correction (Table 2, main text).

	Beta ERS
EASE-1 (cognition and stream of consciousness)	τ = -0.13 (n.s.)
EASE-2 (self-awareness)	**τ = -0.51 p = 0.046***
EASE-3 (bodily experiences)	τ = 0.09 (n.s.)
EASE-4 (demarcation)	τ = 0.27 (n.s.)
EASE-5 (existential reorientation)	**τ = -0.52 p = 0.047***
EASE-10 (comprehensive)	τ = -0.2 (n.s.)
EASE - Total score	τ = -0.2 (n.s.)

References:

1. Pfurtscheller, G., Stancák, A. & Neuper, C. Post-movement beta synchronization. A correlate of an idling motor area? *Electroencephalography and Clinical Neurophysiology* **98**, 281–293 (1996).
2. Pfurtscheller, G. & Neuper, C. Event-related synchronization of mu rhythm in the EEG over the cortical hand area in man. *Neuroscience Letters* **174**, 93–96 (1994).
